# Integrated Stress Response Regulation of Corneal Epithelial Cell Motility and Cytokine Production

**DOI:** 10.1167/iovs.63.8.1

**Published:** 2022-07-08

**Authors:** Hsiao-Sang Chu, Cornelia Peterson, Xitiz Chamling, Cynthia Berlinicke, Donald Zack, Albert S. Jun, James Foster

**Affiliations:** 1 Department of Ophthalmology, National Taiwan University Hospital, Taipei, Taiwan; 2 Graduate Institute of Clinical Medicine, College of Medicine, National Taiwan University, Taipei, Taiwan; 3 Department of Molecular and Comparative Pathology, Johns Hopkins School of Medicine, Johns Hopkins University, Baltimore, Maryland, United States; 4 Department of Ophthalmology, Wilmer Eye Institute, Johns Hopkins University School of Medicine, Johns Hopkins University, Baltimore, Maryland, United States

**Keywords:** ATF4, CHOP, integrated stress response, cornea wound healing

## Abstract

**Purpose:**

To investigate the effect of an active integrated stress response (ISR) on human corneal epithelial cell motility and cytokine production.

**Methods:**

ISR agonists tunicamycin (TUN) and SAL003 (SAL) were used to stimulate the ISR in immortalized corneal epithelial cell lines, primary human limbal epithelial stem cells, and ex vivo human corneas. Reporter lines for ISR-associated transcription factors activating transcription factor 4 (ATF4) and XBP1 activity were generated to visualize pathway activity in response to kinase-specific agonists. Scratch assays and multiplex magnetic bead arrays were used to investigate the effects of an active ISR on scratch wounds and cytokine production. A C/EBP homologous protein (CHOP) knockout cell line was generated to investigate the effects of ISR ablation. Finally, an ISR antagonist was assayed for its ability to rescue negative phenotypic changes associated with an active ISR.

**Results:**

ISR stimulation, mediated through CHOP, inhibited cell motility in both immortalized and primary human limbal epithelial cells. Scratch wounding of ex vivo corneas elicited an increase in the ISR mediators phosphorylated-eIF2α and ATF4. ISR stimulation also increased the production of vascular endothelial growth factor (VEGF) and proinflammatory cytokines. ISR ablation, through CHOP knockout or inhibition with integrated stress response inhibitor (ISRIB) rescued epithelia migration ability and reduced VEGF secretion.

**Conclusions:**

We demonstrate that the ISR has dramatic effects on the ability of corneal epithelial cells to respond to wounding models and increases the production of proinflammatory and angiogenic factors. Inhibition of the ISR may provide a new therapeutic option for corneal diseases in which the ISR is implicated.

The corneal epithelium is a uniquely isolated tissue exposed to a broad range of environmental stressors. These include pathogens, hypoxia and the relatively high partial pressure of oxygen, ultraviolet light, and exposure to environmental pollutants.^1^^–^^3^ Finally, the avascularity of the tissue necessitates indirect metabolic exchange in order to preserve optical clarity.^4^ The purpose of this study was to investigate the behavior of corneal epithelial cells in response to activation of the integrated stress response (ISR). This pathway is a critical signaling system that allows for cells to respond to both intracellular and extracellular stressors to which the corneal epithelium is exposed.^3^^,^^5^^,^^6^ The ISR is the primary method by which cells can adjust the protein translational dynamics in response to signaling through four kinases that are responsive to amino acid and glucose deprivation (general control non-depressible protein 2 [GCN2]), heme deficiency (heme-regulated inhibitor [HRI]), viral infection (double-stranded RNA-dependent protein kinase [PKR]), and accumulation of unfolded protein in the endoplasmic reticulum (PKR-like endoplasmic reticulum kinase [PERK]).^5^^,^^7^ Activation of these kinases results in the phosphorylation of eukaryotic translation initiation factor 2-alpha (eIF2α) at serine 51.^8^ This phosphorylation inhibits the Met-tRNA_i_^Met^ carrier activity of eIF2 via sequestration of eIF2B and effective stalling of cap-dependent protein translation. This shift in the translational landscape is also characterized by the expression of key mediators of the ISR, specifically the transcription factors activating transcription factor 4 (ATF4) and C/EBP homologous protein (CHOP).^6^^,^^9^^,^^10^ The expression of these mediators can lead to phenotypic changes that allow for the cell to either adjust to the stress or trigger apoptosis.^9^

In the context of ophthalmology, ablation of ATF4 manifests as anterior segment dysgenesis and microphthalmia, whereas activation of the ISR has been implicated in several diseases involving the cornea, lens, and retina.^2^^,^^3^^,^^11^ The stratified epithelium of the cornea is critical in protecting the underlying cornea stroma from both the causative agents of cellular stress and their sequelae and in minimizing spread to other areas of the eye and compromising visual function.^1^^,^^12^ We therefore believe that the corneal epithelium is both uniquely exposed to triggers of the ISR while also being constrained in how it can respond to these stressors. Characterization of these responses is critically important to the function of the cornea. Our previous publications have identified activation of the ISR in the cornea of keratoconic patients and have highlighted the effects on cellular phenotype both in vitro and in vivo.^11^^,^^13^^,^^14^ Moreover, there is an extensive body of work regarding the activation of this pathway in the eye and other tissues that warrants further investigation in the corneal epithelium.^2^^,^^3^^,^^15^^,^^16^ The purpose of these studies was to characterize and dissect the response of corneal epithelial cells to ISR challenge. As discussed, the ISR is either directly or indirectly associated with the etiology of numerous corneal processes and diseases that warrant further investigations.

We demonstrate that the ISR induces expression of potentially deleterious cytokines and reduces cell motility in in vitro scratch wound assays. We subsequently demonstrate that pharmacological inhibition and genetic ablation of the ISR results in rescue of scratch closure rates and a decrease in vascular endothelial growth factor (VEGF) production. Our results suggest that inhibition of the ISR may be a therapeutic target in corneal diseases where the ISR pathway is implicated in pathogenesis.

## Methods

### Ethics Statement

All human tissue was obtained under established guidelines related to informed consent for research use of human donor and patient corneas and adhered to the tenets of the Declaration of Helsinki. Corneal rims were obtained from surgeries performed at the Wilmer Eye Institute using a protocol approved by the Johns Hopkins Medicine Institutional Review Board and titled “Phenotypic and Genotypic Analysis of Keratoconus” (IRB00260478, PI Foster Approved 11/6/2020).

### ISR Modulators

Tunicamycin (TUN; #12819S; Cell Signaling Technology, Danvers MA, USA) and SAL003 (SAL; #3657; Tocris Bioscience, Bristol UK) were used as generalized ISR activators. Halofuginone (HF; Selleck Chemicals, Houston, TX, USA), sodium arsenite (ARS; MilliporeSigma, St. Louis, MO, USA), and polyinosinic:polycytidylic acid (poly I:C; InvivoGen, San Diego, CA, USA) were applied as GCN2, HRI, PKR, and PERK agonists to induce specific arms of ISR, respectively. Trans-integrated stress response inhibitor (trans-ISRIB; Tocris Bioscience) was investigated as an ISR inhibitor.

### Cell Line and Ex Vivo Corneal Tissue Preparation

Control human limbal epithelial cells were isolated from healthy cadaveric corneas used for keratoplasty (Supplementary Table S1). Briefly, corneal tissue were isolated from the limbal rims using surgical dissection. Tissue was incubated in 1 mg/mL Dispase II (#17105041; Thermo Fisher Scientific, Waltham, MA, USA) dissolved in KGM-Gold BulletKit (#00192060; Lonza Group, Basel, Switzerland) for 2 hours in a 37°C, 5% CO_2_, humidified tissue culture incubator. Epithelial sheets were then isolated by manual dissection and incubated for 15 minutes in an Accutase solution (#A1110501; Thermo Fisher Scientific). Cells were passed through a 70-µm strainer and seeded on tissue culture dishes coated with laminin (#23017015; Thermo Fisher Scientific). Cells were maintained in KGM-Gold supplemented with Normocin (#ant-nr-1; InvivoGen) until 80% confluent; they were then passaged using Accutase and used for downstream experiments or maintained. All primary human limbal epithelial cells were passage number <3 when used in experiments.

Telomerase-immortalized human corneal epithelial (HCLE) cell lines were grown in KGM-Gold media supplemented with Normocin.^17^ Cells were maintained until 80% confluent and then passaged using Accutase. All experiments were carried out in antibiotic-free KGM-Gold media supplemented with 1.13-mM CaCl_2_ unless otherwise stated, the supplementation of which is necessary for normal cellular movement and behavior.^17^^,^^18^

ATF4-mScarlet nuclear localization signal (NLS; plasmid #115970; Addgene, Watertown, MA, USA) and X-box binding protein 1 (XBP1)-mNeonGreen NLS (plasmid #115971; Addgene) were packaged in replication-deficient retroviral viruses. Briefly, human embryonic kidney cells (HEK293) were transfected with pUMVC (#8449; Addgene), pCMV-VSV-G (#8454; Addgene), and the reporter construct using Lipofectamine 3000 transfection reagent (#L3000001; Thermo Fisher Scientific) per the manufacturers’ instructions.^19^ The media were changed after 24 hours, and viral particles were harvested 48 hours post-transfection. Viral production was assayed by transfection of HCLE cells overnight followed by 500-µg/mL Hygromycin B (#10687010; Thermo Fisher Scientific) selection for 7 days. A subset of remaining cells was then challenged with 100 ng/mL TUN for 16 hours to elicit an active ISR with positive nuclear fluorescence being observed via fluorescent microscopy (Eclipse Ts2R; Nikon, Tokyo, Japan). Individual clones were selected via two rounds of limiting dilution isolation. Monoclonal cell lines were tested for reporter activity as before; they were short tandem repeat and mycoplasma tested (Genetic Resources Core Facility, Johns Hopkins University, Baltimore, MD, USA) and then used for downstream applications.

A CHOP knockout HCLE (CHOP^−/−^) cell line was created via CRISPR/Cas9 genome editing. gRNA targeting the Sac1 restriction site in the second exon of CHOP/DDIT3 was cloned into Addgene PX459 all-in-one plasmid.^20^ HCLE cells were transfected with the plasmid and Lipofectmine STEM reagent (#STEM00001; Thermo Fisher Scientific). Cells were selected for puromycin resistance over 4 days and then clones were picked and subjected to two rounds of limiting dilution isolation. Editing was confirmed by loss of the Sac1 restriction site in PCR product, and loss of function was confirmed by western blot and immunofluorescence (Supplementary Fig. S1).

Intact corneas were harvested from cadaverous whole globes within 24 hours postmortem (Lions Vision Gift; Supplementary Table S1). Corneas were bisected, with one half being saved for time 0. Epithelium of the central cornea of the right eye was debrided with a scalpel, and the contralateral cornea was used as a control. Corneas were washed in PBS and then immersed in calcium-supplemented KGM-Gold overnight. Corneas were then fixed, cryosectioned, and stained for phosphorylated eIF2α (p-eIF2α) and ATF4 as below.

### alamarBlue Reduction/Cell Survival Assay

The alamarBlue/resazurin reduction assay (#R7017; MilliporeSigma) was used to quantify total cell numbers. A working concentration of 55 µM resazurin was prepared in PBS, and at each media change, 1/10 volume was applied to the well followed by incubation at 37°C for 50 minutes. Fluorescence was then measured on a fluorimeter with 540-nm excitation and 590-nm emission, and each well was read in duplicate (Safire 2; Tecan Trading, Männedorf, Switzerland). Cell numbers were derived from standard curves of known cell numbers.

### Immunohistochemistry

HCLE cells were plated on eight-well chamber slides and grown in KGM-Gold overnight; cells were then dosed with ISR modulators or vehicle control. Cells were washed in PBS and fixed in 4% paraformaldehyde in PBS for 15 minutes. They were then permeabilized in 0.1% Triton X-100 in PBS for 5 minutes. Slides were blocked in 3% fetal bovine serum and 2% normal goat serum in PBS for 1 hour. Primary antibody was diluted in blocking buffer 1:200 and incubated overnight at 4°C. After washing for 15 minutes in PBS, secondary antibody was diluted 1:500 in blocking buffer and incubated for 1 hour at room temperature. F-actin was visualized with Phalloidin, DyLight 488, or DyLight 650 (Thermo Fisher Scientific) diluted 1:200 in blocking buffer and incubated for 15 minutes. Nuclei were visualized with 4′,6-diamidino-2-phenylindole (DAPI). Images (10×) were obtained on an Axio Imager.A2 (Carl Zeiss Meditec, Jena, Germany) utilizing AxioVision software. High-magnification images were obtained on a Zeiss LSM 510 Meta confocal microscope with a 63× objective utilizing Zen software.

### Western Blot

Proteins were detected using standard western blotting techniques. In brief, cells were solubilized in radioimmunoprecipitation assay buffer with protease and phosphatase inhibitors (Roche, Basel, Switzerland) then collected using a cell scraper. The samples were then sonicated, and protein concentrations were recorded using the BCA Protein Assay Kit (#71285; MilliporeSigma). For quantification of ATF4, eIF2, p-eIF2, CHOP, and glyceraldehyde 3-phosphate dehydrogenase (GAPDH), 15 µg of total protein per lane was run on 10% gradient gel (Bio Rad Laboratories, Hercules, CA, USA). Proteins were then transferred to polyvinylidene fluoride membranes using a wet transfer apparatus and then blocked with 5% non-fat dry milk or bovine serum albumin in PBS or Tris-buffered saline (TBS) for 1 hour at room temperature. Primary antibodies were diluted as appropriate (Supplementary Table S2) in blocking buffer with 0.1% Tween 20 and incubated at 4°C overnight. Membranes were washed 15 minutes three times (0.1% Tween 20 in PBS or TBS). Horseradish peroxidase-conjugated secondary antibodies were diluted 1:5000 in blocking buffer and incubated for 1 hour at room temperature. Chemiluminescent substrate was then applied to the membrane and imaged on a commercial system (Invitrogen iBright CL750 Imaging System; Thermo Fisher Scientific). Protein densitometry was determined relative to GAPDH using ImageJ.^21^

### Multiplex Bead Array and ELISA

Multiplex cytokine profiling was carried out utilizing the MILLIPLEX Human Cytokine/Chemokine immunoassay (#HCYTA-60K-PX48; Merck, Damstadt, Germany). In brief, on the day of analysis, frozen (–20°C) supernatant collected from ISR modulator-treated cells or controls was quickly thawed. Forty-eight cytokines (Supplementary Table S3) were measured by an automated Luminex Bio-Plex 200 IS system immunoassay analyzer (Bio Rad Laboratories) that performs automated chemiluminescent immunoassays. We analyzed cytokines using commercially available magnetic microsphere beads (MilliporeSigma). Microsphere beads of defined spectral properties and conjugated to antibodies directed against each cytokine were pipetted into 96-well plates. Approximately 2500 antibody-conjugated microspheres per cytokine were added to each well. The protocol for these assays involves mixing 50 µL undiluted supernatant with 25 µL antibody-coated microspheres, overnight incubation in the dark at 4°C, washing three times with wash buffer, adding detection antibody, and incubation for 1 hour at room temperature. We then added 25 µL Streptavidin, Phycoerythrin Conjugate solution in the well to form a capture sandwich immunoassay followed by incubation in the dark for 30 minutes at room temperature, washing three times with washing buffer, and then the addition of 150 µL sheath fluid. Microspheres were aspirated through the flow cell of a dual laser Luminex 200 instrument (Luminex Corporation, Austin, TX, USA). Median fluorescence intensities of microspheres specific for each cytokine were recorded for each well to calculate the cytokine concentration by 7-point logistic regression. Cell supernatant interleukin (IL)-6 and VEGF were confirmed by conventional ELSIA using ELISA Max Deluxe Set Human IL-6 (#430504; BioLegend, San Diego, CA, USA) and ELISA MAX Deluxe Set Human VEGF (#446504; BioLegend) according to the manufacturer's instructions.

### Quantitative PCR

RNA was extracted from cells using RNEasy (QIAGEN, Hilden, Germany), and reverse transcription was carried out using the iScript cDNA Synthesis Kit (Bio Rad Laboratories) per the manufacturer's instructions. A total of 15 ng cDNA was subjected to qPCR using TaqMan chemistry on the CFX384 Touch Real-Time PCR system (Bio Rad Laboratories). Primers were obtained from Thermo Fisher Scientific (VEGF, #Hs00900055_m1; CHOP, #Hs01090850_m1). Each test was run in triplicate with relative transcript levels of target gene expressed as 2^-ΔΔCT^ with *POLR2A* (#Hs00172187_m1; Thermo Fisher Scientific) as the housekeeping control gene.

### Statistical Analysis

Quantitative data are presented as mean ± SEM and were analyzed for statistical significance by unpaired, two-tailed Student's *t-*test or 1-way ANOVA using Prism (GraphPad; San Diego, CA, USA). Significance is presented as *P < 0.05, **P < 0.01, ***P < 0.001, and ****P < 0.0001.

## Results

### Induction of the ISR in HCLE Cell Lines

**Figure 1.**
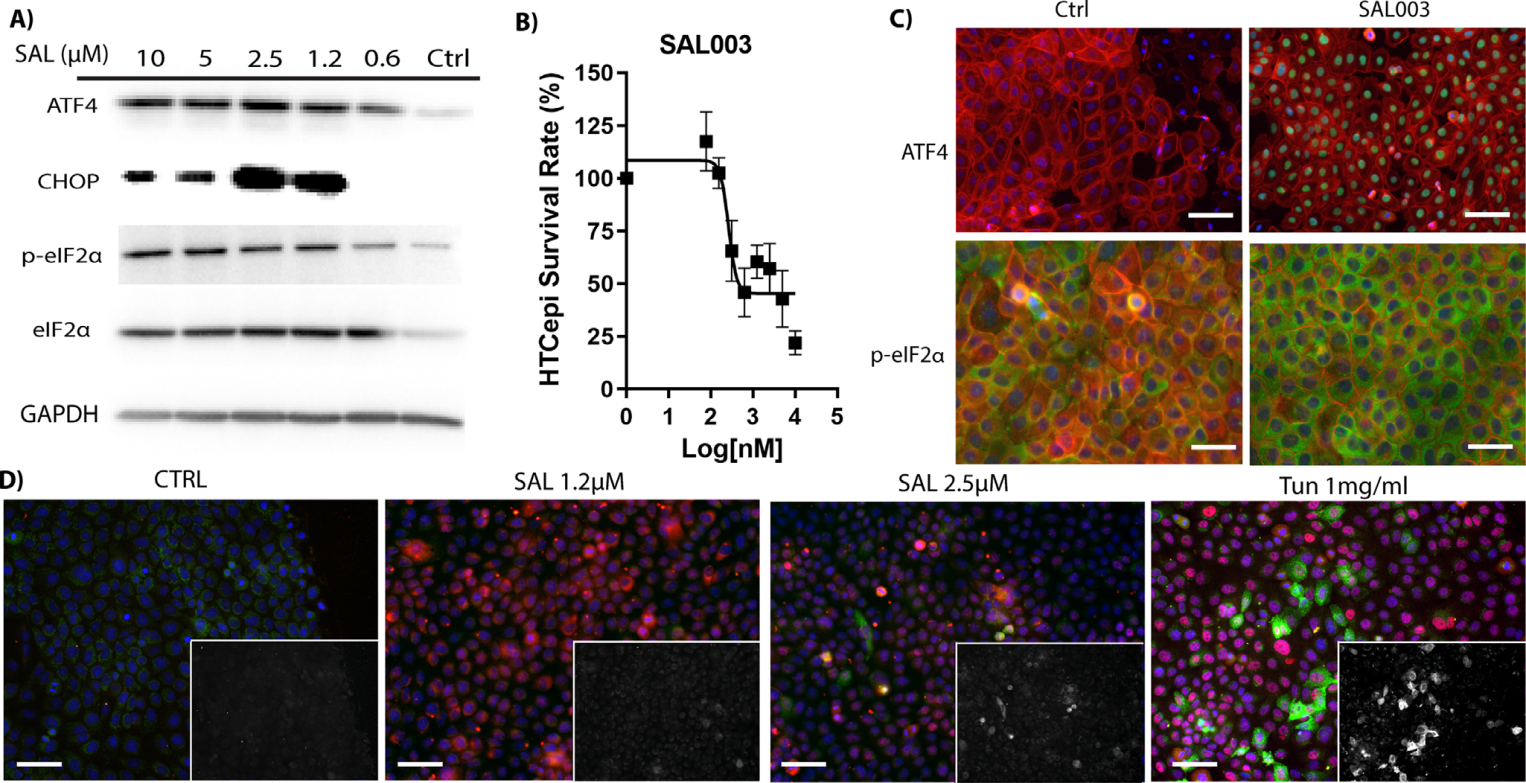
ATF4 induction with SAL in immortalized HCLE cells. (**A**) Western blots of ISR mediators ATF4, CHOP, and p-eIF2α in response to SAL 16-hour challenge. (**B**) HCLE toxicity as determined by alamarBlue reduction in response to SAL challenge after 48 hours (*n* = 4). (**C**) ATF4 and p-eIF2α induction in HCLE cell lines after incubation with the ISR agonist SAL (2.5 µM). Protein of interest, *green*; F-actin, *red*; DAPI, *blue*. *Scale bar*: 50 µm. (**D**) CHOP (*red*) and cleaved caspase 3 (inlay, *green*) induction in response to overnight ISR challenge. *Blue*, DAPI. Toxic levels of TUN (1 mg/mL) were used to induce cell death (active cleaved caspase 3). *Scale* *bar*: 100 µm.

Based on our previous publications, we stimulated the ISR using the salubrinal derivative SAL, an inhibitor of the phosphatases growth arrest and DNA-damage inducible protein 34 (GADD34) and constitutive repressor of eIF2α phosphorylation (CReP), for 48 hours.^13^^,^^22^^,^^23^ A concentration of 2.5 µM was shown to activate the ISR (Fig. 1A) as determined by the expression of ATF4 and CHOP and increased p-eIF2α/eIF2α ratio. We then sought to identify a concentration of SAL that would activate the ISR but not trigger apoptosis. We utilized the alamarBlue reduction assay to identify the lethal concentration 50% (LC_50_) of SAL; 10.45 µM was identified as the LC_50_, with 2.5 µM not affecting cell number. This demonstrated that it is possible to maintain cells in a long-term state of ISR activation without inducing apoptosis in vitro (Fig. 1B). Immunofluorescence demonstrated nuclear expression of ATF4 and apparent increase in p-eIF2α signal in treated cells after 16-hour treatment with 2.5-µM SAL (Fig. 1C, Supplementary Fig. S2), which was used to confirm ISR activation. As activation of the ISR can cause cell death, we ensured that the selected treatment concentrations did not induce apoptosis. We conducted immunofluorescent staining of the ISR effector CHOP and apoptosis marker cleaved caspase 3 in response to overnight ISR challenge. The SAL challenge at 1.25 µM and 2.5 µM elicited a strong CHOP response (red) in agreement with Figure 1A. However, there was no corresponding increase in cleaved caspase 3 activity (green) in these conditions, whereas the 1-mg/mL TUN (known to be toxic) did elicit cleaved caspase 3 activity (Fig. 1D, Supplementary Fig. S3).

### ATF4 Induction in Response to eIF2α Kinase Agonists

**Figure 2.**
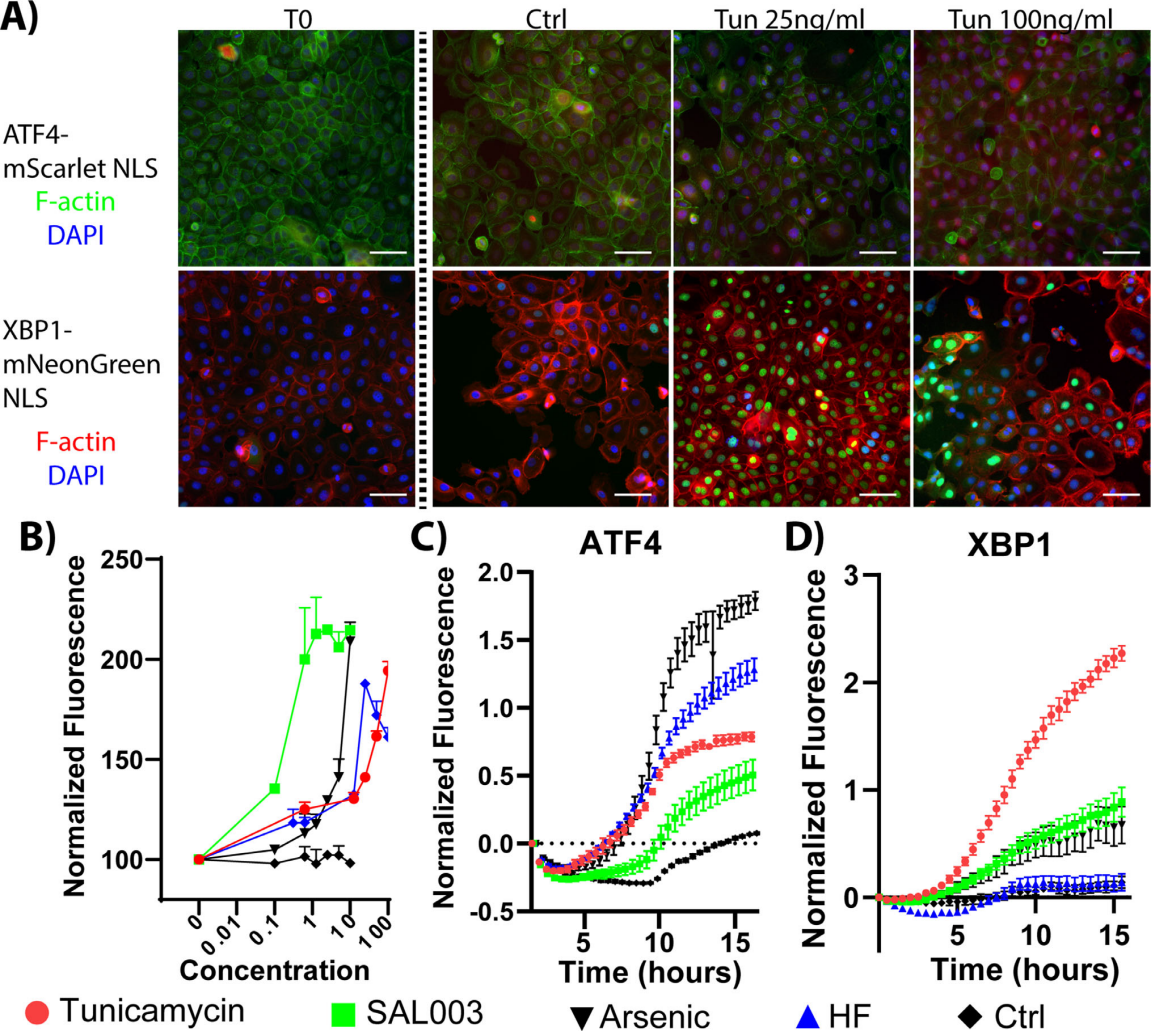
ISR induction with specific eIF2α kinase agonists. (**A**) Fluorescent images of ISR-responsive cell line activity at time 0 (T0), at 16 hours (Ctrl), and in response to TUN treatment at 25 ng/mL and 100 ng/mL. Both cell lines demonstrated strong nuclear staining (ATF4, *red*; XBP1, *green*) and F-actin stained with phalloidin (ATF4, *green*; XBP1, *red*). (**B**) Relative fluorescent intensity of ATF4–NLS reporter line in response to ISR agonist challenge. TUN (ng/mL), SAL (µM), ARS (µM), and HF (nM). (**C**) ATF4 reporter line fluorescence activity in response to ISR stimuli; fluorescent readings were recorded every 15 minutes for 16 hours. (**D**) XBP1 reporter cell line in response to ISR stimuli.

As discussed previously, several stressors can activate the ISR. Monoclonal ATF4–mScarlet NLS and XBP1–mNeonGreen NLS reporter HCLE cell lines were generated to facilitate direct observation of ISR and unfolded protein response (UPR) activity.^24^ Induction of reporter gene expression was validated through visualization after 16-hour exposure of 25-ng/mL and 100-ng/mL TUN (Fig. 2A). Next, dose-dependent and temporal induction of ATF4 signal was monitored in response to ISR-stimulating agonists. Serial dilutions of agonists starting from SAL (10 µM) and TUN (5 µg/mL) were tested for their ability to stimulate ATF4 reporter line activity. We also examined the ability of agonists upstream of eIF2α to induce reporter activity. Halofuginone (HF; 100 nM), an inhibitor of prolyl-tRNA synthetase and a small molecule agonist of GCN2, and arsenic (ARS; 6 µM), an activator of HRI and the UPR, were tested for their ability to induce ATF4 reporter activity with 16-hour incubation (Fig. 2B). All prospective agonists were shown to increase reporter activity after incubation. TUN (25 ng/mL), SAL (2.5 µM), HF (25 nM), and ARS (3 µM) were subsequently selected as the optimum agonist concentrations for induction of reporter activity. We then sought to investigate whether there were changes in the rates of induction of reporter activity in response to these agonists. TUN, HF, and ARS were observed above baseline at 8 hours, and SAL activity increased above baseline at 10 hours (Fig. 2C). Specificity of the agonists was confirmed with the XBP1 reporter line, which did not respond to GCN2 activity stimulated through HF (Fig. 2D) but did increase in response to agonists of PERK (Supplementary Fig. S4).

### ISR Activity in Scratch Assays

**Figure 3.**
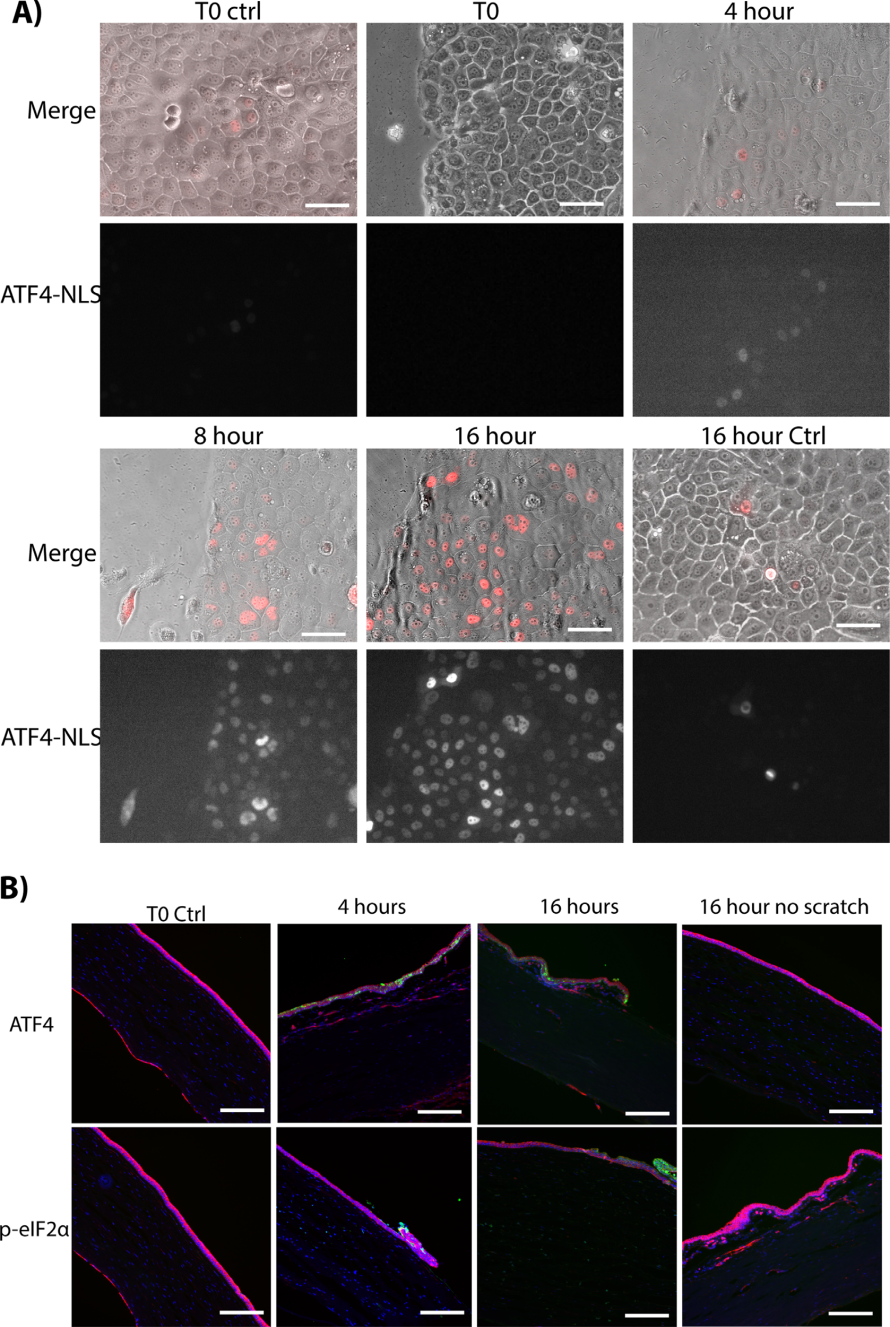
ISR activity in scratch assays. (**A**) Merged brightfield and fluorescent (*red*) images of ATF4–NLS activity in scratch assays. The unscratched control (Ctrl) wells (T0); immediately after scratch (T0); at 4, 8, and 16 hours after scratching; and 16-hour Ctrl show baseline levels of ATF4–NLS activity. Isolated fluorescent images indicate the intensity and localization of the signal. *Scale* *bar*: 50 µm. (**B**) ATF4 and p-eIF2α (*green*) staining of ex vivo human cornea in response to corneal epithelial debridement. F-actin, *red*; DAPI, *blue*. Initial levels at the scratch edge at 4 hours and 16 hours after scratching are shown. *Scale* *bar*: 200 µm.

We examined whether the ISR was affected in response to in vitro wound assays (Fig. 3A). Scratch assays were performed on the ATF4 reporter cell line, and fluorescent activity was observed over the duration for response to wounding. At 4 hours after wounding, ATF4 reporter activity was observed in cells close to the scratch edge; by 8 hours this had progressed to 100 µm from the edge. At 16 hours after scratch, most cells within 300 µm of the edge demonstrated ATF4 reporter activity. Activity in the unscratched control (Ctrl) wells was negligible at time 0 and at 16 hours. Activity of the XBP1-sp reporter cell line also demonstrated increased activity in response to scratch (Supplementary Fig. S2).

We then sought to evaluate whether the ISR activation could be elicited in ex vivo human corneas in response to wounding (Fig. 3B). ATF4 and p-eIF2α (green) were both detected in the scratched cornea at 4 hours and 16 hours after wounding. Importantly, this was not observed in either the T0 control or the unscratched control.

### ISR Activation Delays Wound Healing

**Figure 4.**
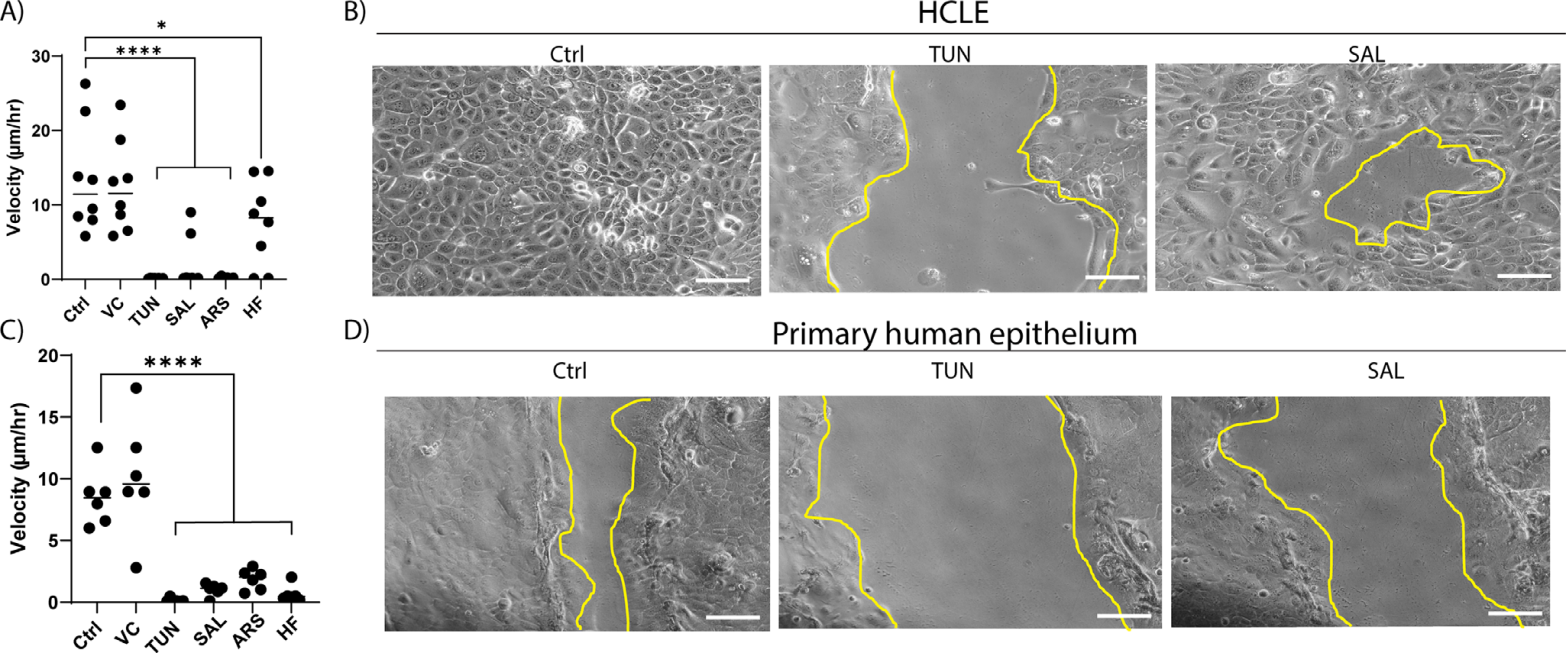
ISR inhibition of corneal epithelial motility. (**A**) Scratch closure velocity in HCLE cells measured over 16 hours in response to ISR agonists. Ctrl, control; VC, vehicle. TUN, 25 ng/mL; SAL, 2.5 µM; ARS, 3 µM; HF, 25 nM (*n* = 8). ****P < 0.0001, *P = 0.039. (**B**) Representative images of HCLE cells after scratch assay showing decreased closure in response to ISR agonist at 16 hours. (**C**) Primary human limbal epithelial closure velocity in response to ISR challenge (*n* = 6). (**D**) Representative images of decreased closure velocity in primary human limbal epithelial cells. The *yellow line* indicates the leading edge of the scratch. *Scale* *bar*: 100 µm.

To investigate the potential impact of the ISR on cell migration, scratch assays were performed on both immortalized HCLE cell lines (Figs. 4A, 4B) and primary human limbal epithelial cell lines (Figs. 4C, 4D). Cells were pretreated with agonists for 6 hours before being scratched. HCLE control (Ctrl) and vehicle control (VC) cells closed the wound at 13.50 ± 2.60 µm/h and 12.50 ± 2.20 µm/h, respectively. In response to the agonist challenges, HCLE closure velocity was reduced: TUN (25 ng/mL), 0.13 ± 0.01 µm/h; SAL (2.5 µM), 2.01 ± 1.20 µm/h; ARS (3 µM), 0.21 ± 0.05 µm/h (all, *P <* 0.0001); and HF (25 nM), 7.60 ± 2.00 µm/h (*P <* 0.05).

Primary human limbal epithelium also showed decrease of scratch healing velocity in response to ISR challenge, and the Ctrl and VC velocities (8.50 ± 2.30 µm/h and 10.13 ± 1.95 µm/h, respectively) were comparable to those for HCLE cultures. However, the other agonists were all significantly slower: TUN, 0.15 ± 0.06 µm/h; SAL, 1.00 ± 0.34 µm/h; ARS, 1.84 ± 0.34 µm/h; and HF, 0.65 ± 0.27 µm/h (all *P <* 0.0001). This suggests that control of cell motility dynamics is mediated, at least in part, through the ISR.

### ISR Activation Induces Angiogenic and Proinflammatory Cytokine Production

**Figure 5.**
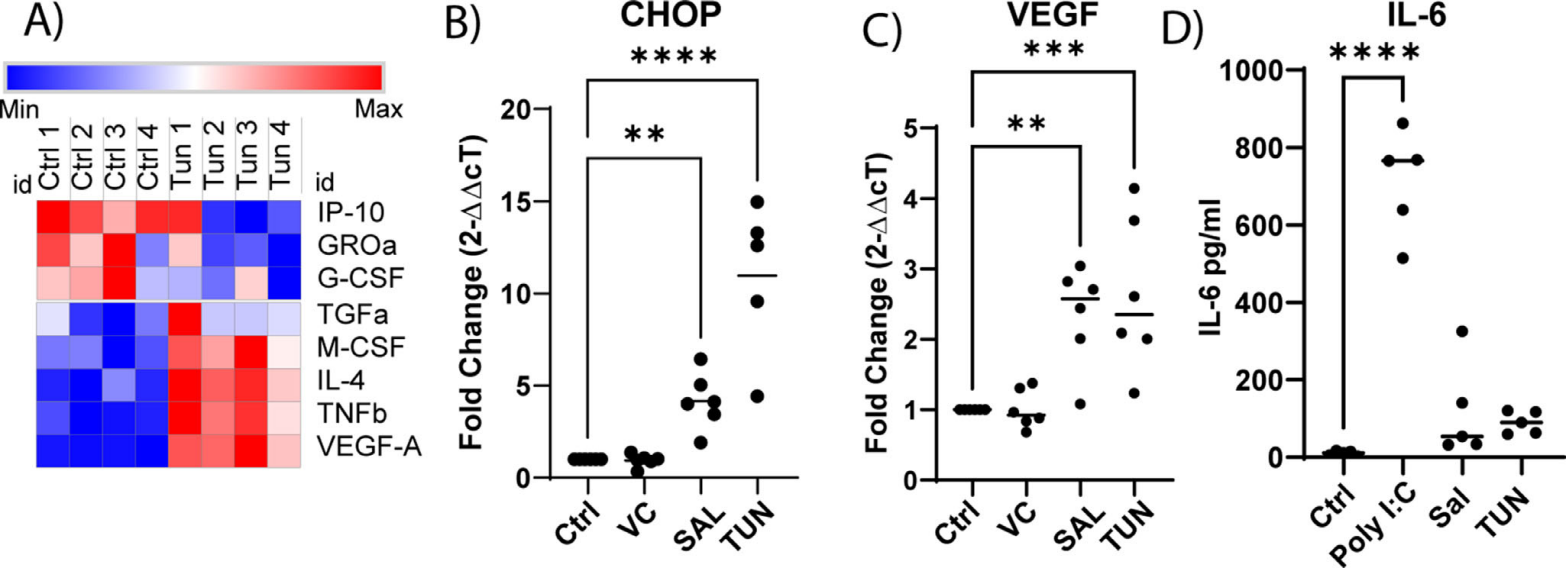
Cytokine production in response to ISR stimulation. (**A**) Most changed cytokine production of HCLE in response to TUN (25 ng/mL) challenge as determined by multiplex bead assay. (**B**) qPCR of CHOP induction in response to SAL (2.5µM) and TUN (25 ng/mL) challenge (*n* = 6). (**C**) qPCR of VEGF-A in response to SAL (2.5 µM) and TUN (25 ng/mL) challenge (*n* = 6). (**D**) IL-6 production as determined by ELISA in response to ISR challenge; poly I:C (200 ng/mL) was used as a positive control (*n* = 4).

In addition to investigating the effect of ISR on cell motility, we also investigated the effect of an active ISR on cytokine secretion. Utilizing a multiplex magnetic bead assay, we assayed the production of cytokines in response to 16-hour TUN (25 ng/mL) challenge. Of the 48 cytokines assayed, three were consistently downregulated and five were consistently upregulated (Fig. 5A). Other cytokines were not included, as they were not significant (less than twofold change) or were not detected in all samples (Supplementary Table S4). Of the upregulated cytokines, VEGF, TNFβ, and IL-4 were the most consistently upregulated. CHOP (DDIT3), a key effector of the ISR, was assayed as a marker for ISR activation and was induced in response to 2.5-µM SAL (4.1 ± 0.62; *P <* 0.01) and 25-ng/mL TUN (10.96 ± 1.84; *P <* 0.0001) (Fig. 5B). This corresponded to increased transcription of VEGF in response to 2.5-µM SAL (2.35 ± 0.29; *P <* 0.01) and 25-ng/mL TUN (2.62 ± 0.44; *P <* 0.001) stimuli (Fig. 5C). IL-6 was assayed via conventional ELISA, as it has been shown to be involved in corneal wound healing (Fig. 5D), despite increasing from 10.27 ± 2.7 pg/mL in Ctrl to 117 ± 55.72 pg/mL in 2.5-µM SAL–treated cells and 90.02 ± 12.89 pg/mL in 25-ng/mL TUN–treated cells; this was not considered significant when compared with the obligate IL-6 agonist poly I:C (710 ± 60 pg/mL; *P <* 0.0001).

### CHOP Controls Cellular Response to the ISR

**Figure 6.**
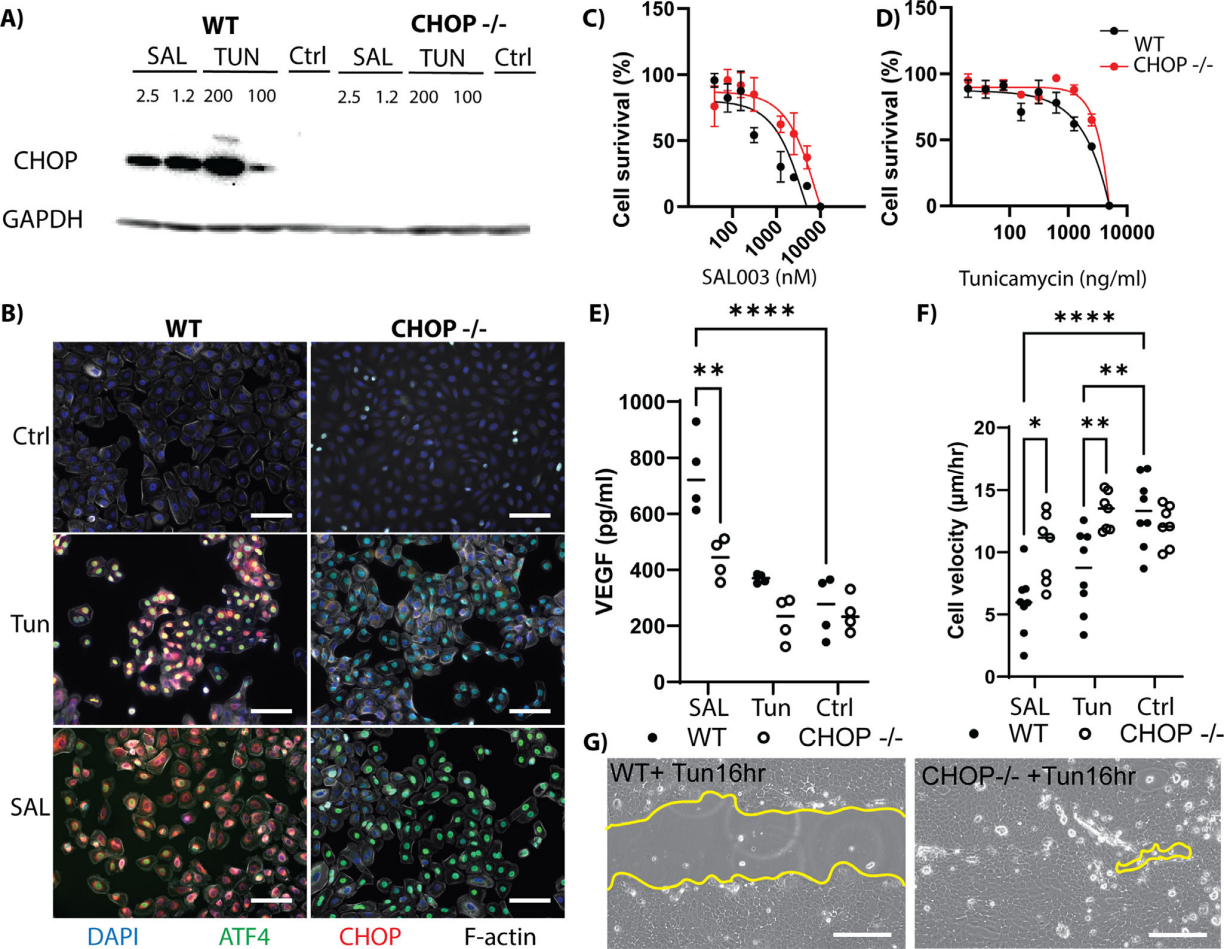
Response to ISR challenge by CHOP knockout HCLE. (**A**) Western blot of CHOP in WT and knockout (CHOP^−/−^) HCLE cell lines in response to ISR challenge with SAL (2.5 and 1.25 µM) and TUN (200 and 100 ng/mL) for 16 hours, with GAPDH as the loading control. (**B**) Nuclear localization of ATF4 (*green*) and CHOP (*red*) in response to 16-hour ISR stimulation with TUN (25 ng/mL) or SAL (2.5 µM). F-actin is shown in *gray*, and DAPI is shown in *blue*. *Scale* *bar*: 50 µm. (**C**) The alamarBlue cell survival assay showed that the CHOP^−/−^ cell line was more resistant to SAL (LC_50_ = 15 µM) than the WT cell line (LC_50_ = 6 µM) at 24 hours. (**D**) The CHOP^−/−^ cell line was more resistant to TUN (LC_50_ = 5 µg/mL) than the WT cell line (LC_50_ = 1 µg/mL) at 24 hours. (**E**) VEGF production as determined by ELISA in response to 16-hour ISR challenge with SAL (2.5 µM) and TUN (25 ng/mL). WT is shown as *solid circles*, knockout (CHOP^−/−^) is shown as *hollow circles* (*n* = 4). (**F**) Scratch closure velocity of WT (*solid circle*) and knockout (CHOP^−/−^; *hollow circle*) HCLE cell lines in response to ISR challenge with SAL (2.5 µM) and TUN (25 ng/mL) (*n* = 8). (**G**) Representative images of closure after 16 hours of exposure to TUN (25 ng/mL). The *yellow line* indicates the leading edge of the scratch. *Scale bar*: 100 µm.

The transcription factor CHOP is the ultimate effector of cell fate outcomes in response to an active ISR. To investigate whether CHOP was ultimately responsible for the negative phenotypic effects associated with the ISR we generated a CHOP knockout immortalized epithelial cell line. The absence of CHOP was confirmed by stimulation with either SAL (2.5 or 1.25 µM) or TUN (200 or 100 ng/mL) for 16 hours, and no detectable expression of CHOP was observed (Fig. 6A). Immunofluorescence demonstrated that CHOP was not detectable even with strong ATF4 nuclear expression (Fig. 6B). Cell survival was assayed as before, and the CHOP^−/−^ cell line was shown to be more resistant to the pro-apoptotic concentrations of the agonists than wild-type (WT), with LC_50_ values of 15 µM and 6 µM, respectively, for SAL (Fig. 6C) and 5 µg/mL and 1 µg/mL for TUN (Fig. 6D) at 24 hours. Expression of VEGF was also found to be decreased in CHOP^−/−^ cells in response to TUN and SAL challenge (Fig. 6E). SAL (2.5 µM) elicited the production of 745 ± 71 pg/mL in WT compared to 438 ± 36 pg/mL in CHOP^−/−^ cells (*P <* 0.01). WT cells stimulated by TUN (25 ng/mL) produced 368 ± 7 pg/mL, whereas the CHOP^−/−^ cells expressed 221 ± 39 pg/mL, which was nearly identical to the control levels of WT cells (265 ± 55 pg/mL) (*P* > 0.05).

Depressed cell motility was also rescued in CHOP^−/−^ cells (Figs. 6F, 6G); WT cells closed the scratch at 13.29 ± 2.8 µm/h and then slowed to 5.88 ± 2.5 µm/h and 8.43 ± 3.3 µm/h in the presence of SAL (2.5 µM) and TUN (25 ng/mL), respectively (*P <* 0.0001 and *P <* 0.01, respectively). However, in the CHOP^−/−^ cells, unchallenged velocity was 12.14 ± 1.6 µm/h, SAL velocity was 10.27 ± 2.7 µm/h, and TUN velocity was 13.28 ± 1.5 µm/h, suggesting that CHOP was responsible for regulating the response to ISR challenge rather than the upstream challenge itself.

### Pharmacological Inhibition of the ISR Recapitulates CHOP^−/−^ Phenotype

**Figure 7.**
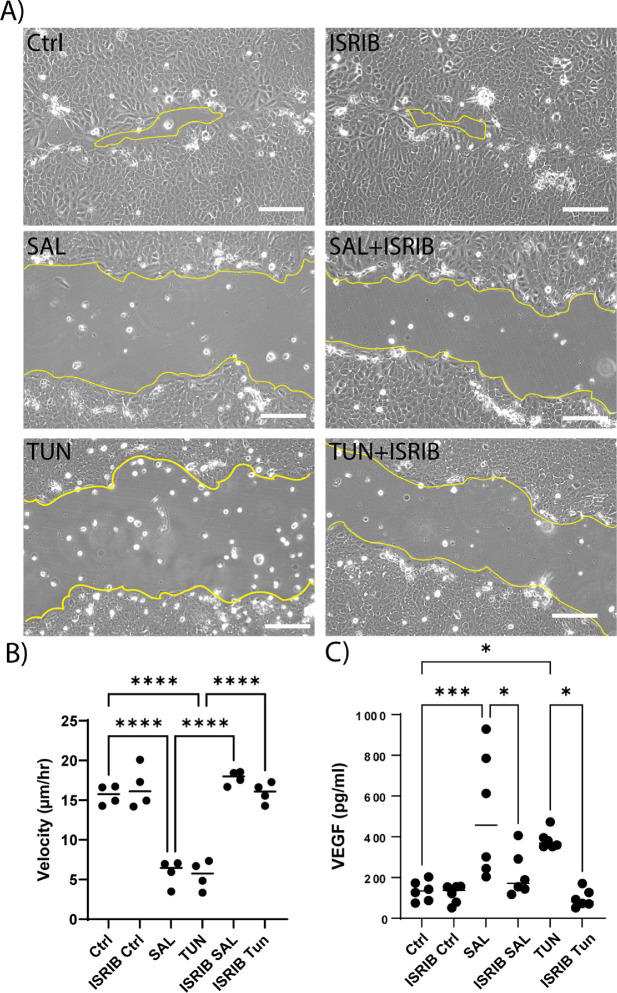
Small molecule rescue of ISR phenotype with ISRIB. (**A**) Representative images of HCLE cell line during scratch assays. Ctrl and ISRIB are comparable; SAL (2.5 µM) and TUN (25 ng/mL) showed slower closing velocity. The 2-hour pretreatment with ISRIB showed increased closure velocity (ISRIB + SAL/ISRIB + TUN). The *yellow line* indicates the leading edge of the scratch. *Scale* *bar*: 100 µm. (**B**) Scratch closure velocity quantification in unchallenged (Ctrl, ISRIB Ctrl), ISR challenged (SAL/TUN), and ISRIB pretreated and ISR challenged + ISRIB (ISRIB + SAL/ISRIB + TUN) HCLE cells (*n* = 4). (**C**) VEGF production over 16 hours in response to ISR challenge (SAL, 2.5 µM; TUN, 25 ng/mL), ISRIB alone (20 ng/mL), or pretreatment and challenge + ISRIB (ISRIB + SAL/ISRIB + TUN) (*n* = 6).

The small molecule ISRIB has been demonstrated as an effective inhibitor of ISR-stimulated effects in other cell types and disease models.^13^^,^^25^ We sought to investigate whether ISRIB would decrease or rescue the deleterious phenotype caused by ISR activation. ISRIB acts downstream of eIF2α and holds the eIF2B decamer together, increasing its stability and thus its ability to participate in translation.^26^ We pretreated HCLE cells for 2 hours with 20-nM ISRIB and then challenged the cells with either TUN (25 ng/mL) or SAL (2.5 µM) for 6 hours before scratching and monitoring scratch closure velocity (Figs. 7A, 7B). Unchallenged HCLE cells closed the scratched area at 15.63 ± 0.6 µm/h, and ISRIB alone closed it at 16.63 ± 1.3 µm/h; when challenged with SAL and TUN, cells significantly slowed their closure velocity to 5.86 ± 0.6 µm/h and 5.59 ± 0.8 µm/h, respectively (*P <* 0.0001 and *P <* 0.01, respectively) (Fig. 7B). In the ISRIB pretreated cells, the closure rate was comparable to that of the control cells, with velocities of 17.8 ± 0.4 µm/h and 15.9 ± 0.6 µm/h in SAL- and TUN-challenged cells, respectively. Finally, we evaluated whether VEGF production was attenuated with ISRIB. As before, cells were pretreated for 2 hours in 20-nM ISRIB followed by 16-hour incubation with SAL/TUN ± ISRIB. VEGF production increased in response to ISR stimulation from 134 ± 20 pg/mL in control cells to 512 ± 120 pg/mL in SAL-treated cells and 385 ± 18.8 pg/mL in TUN-treated cells (*P <* 0.001 and *P <* 0.05, respectively) (Fig. 7C). In pretreated cells, this induction was decreased to 217 ± 45 pg/mL and 97 ± 18 pg/mL, respectively, in response to SAL and TUN ISR challenge, rates that are comparable to those of the control.

## Discussion

We have demonstrated that activation of the ISR imparts a deleterious phenotype on corneal epithelial cells in vitro*.* This is characterized by decreased motility, increased expression of VEGF, and an increase in proinflammatory cytokines. This phenotype can be abrogated through deletion of the ISR effector CHOP, demonstrating that this transcription factor is required for the unwanted response to ISR challenge. Importantly, this phenotype appears to be a potential therapeutic target with pharmacological intervention with ISRIB that is sufficient to inhibit ISR-stimulated VEGF expression and decreased wound healing velocity. The ISR has been attributed directly or implicated in the pathogenesis of multiple diseases, although those of the cornea have been overlooked. This is despite the observation that the corneal epithelium is exposed to all of the numerous environmental stimuli that can trigger the ISR, and we have identified the pathways activated in keratoconus stromal and epithelial cells.^11^ In the absence of either a reported keratoconic epithelial cell line or a widely accepted model of keratoconus, we initiated an investigation into the effects of pathway activation and abrogation on corneal epithelial cells in vitro as a prelude to further characterization of the pathway in patients.

In this article, we have reported that the PERK/ATF4/CHOP cascade produces a potentially deleterious phenotype with characteristics that are common to several ophthalmic pathologies in which the ISR is known to be or is potentially involved.^2^^,^^3^^,^^13^^,^^27^^–^^29^ The ISR can be triggered by multiple independent factors and to different extents, depending on the nature and duration of signal. These factors include hypoxia, glucose deprivation, oxidative stress, viral infection, altered fat or cholesterol concentrations, and genetic mutations.^10^^,^^30^ Although it is important to note that the oligomerization of PERK is only one arm of the ISR, in this context we believe it is both the most relevant to the corneal surface and the most amenable to therapeutic intervention. As previously detailed, multiple agonizing and antagonizing small molecules exist to target distinct arms of the ISR.^3^ By characterizing the response in vitro to these potent agonists, we believe that we have shown that this pathway is a potential contributor to corneal dysfunction.

Here, we utilized tunicamycin for its well-characterized ability to agonize the ISR and the substantial body of work documenting its intracellular effects.^31^^,^^32^ We also utilized the salubrinal analog SAL003 to stimulate a non-specific, kinase-independent increase in ISR.^10^ This enabled us to ensure that any deleterious effects were specific to the downstream signaling of PERK rather than non-canonical signaling through other signaling cascades of the UPR.^33^

Although the transcription factor ATF4 is known to be a key regulator of the ISR, the ultimate effector of the ISR is the transcription factor CHOP (as it is downstream of ATF4).^9^ CHOP knockout mice have no overt ophthalmic phenotype, but they do have metabolic disorders and issues with resolution of the UPR.^34^^,^^35^ In the cornea, CHOP has been implicated in the pathogenesis of Fuchs’ dystrophy and diabetic keratopathy, and ATF4 has been demonstrated in ocular surface infection and keratoconus, as well as during the lens fiber development that is required to stimulate anterior segment development.^3^^,^^16^^,^^34^^,^^36^ The results presented here demonstrate that the ISR, particularly CHOP, has a crucial role in coordinating the response to an active ISR. We have demonstrated that in corneal epithelial cells an active ISR inhibits cell migration and stimulates VEGF production. Interestingly, previous studies report induction of neoplastic cell migration by other ISR stimulators.^37^^,^^38^ We believe that these differential responses are due to the “sheet-like” movement of the corneal epithelium compared to the individual cell motility exhibited by many cancerous cell lines.^39^^,^^40^ Further studies will also investigate whether activation of the pathway in animal models of specific diseases is amenable to ISR intervention, as here we have discussed only the pathway effects in vitro; however, these results are concordant with previous publications linking the ISR with increased VEGF expression in the retina and in cancer.^41^^,^^42^ Perhaps the most striking result is the demonstration that CHOP regulates VEGF production in corneal epithelial cell lines and that the response is attenuated with ISR inhibition. Corneal neovascularization has been attributed to elevated VEGF, and, accordingly, the standard of treatment involves steroids and anti-VEGF therapies.^43^ Potential risks of these treatments include infection, glaucoma, cataracts, and weakening of the cornea, and they have variable efficacy. Our findings suggest that targeting of CHOP and the ISR through small molecules such as ISRIB may yield beneficial results and a new opportunity in corneal neovascularization therapy.

## Conclusions

The ISR is a powerful signaling pathway that is known to play a key role in coordinating protein dynamics and response to cellular stress. We demonstrate that, beyond this, the pathway can regulate cell motility and cytokine production in the corneal epithelium. Crucially, this pathway is amenable to small-molecule intervention; thus, targeting the ISR may be considered as a therapeutic strategy for diseases in which angiogenesis or delayed wound healing is contributory.

## Supplementary Material

Supplement 1
