# Supplementary material for: Integrated Stress Response Regulation of Corneal Epithelial Cell Motility and Cytokine Production
Source: Invest Ophthalmol Vis Sci. 2022 Jul 8;63(8):1. doi: 10.1167/iovs.63.8.1 (PMC9279922; doi:10.1167/iovs.63.8.1)
Supplement: Supplement 1 [file iovs-63-8-1_s001.pdf]

Glossary (From A to Z):

ATF4: activating transcription factor 4

ARS: Sodium arsenite

cAMP: cyclic adenosine monophosphate

CCT: CCT020312

C/EBP: CCAAT/enhancer binding proteins

CHOP: C/EBP homologous protein

CReP: constitutive repressor of eIF2α phosphorylation

eIF2: eukaryotic translation initiation factor 2

GADD34: growth arrest and DNA-damage inducible protein 34

GCN2: general control non-depressible protein 2

GCSF: granulocyte colony-stimulating factor

GRO α **:** growth related oncogene alpha

HF: Halofuginone

HRI: heme-regulated inhibitor / heme-regulated eIF2α kinase

IL: interleukin

IP-10: interferon gamma-induced protein 10, also know as C-X-C motif chemokine ligand 10 (CXCL10)

ISR: integrated stress response

ISRIB: integrates stress response inhibitor

met-tRNA: methylated-transfer RNA

PERK: PKR-like endoplasmic reticulum kinase

PKR: protein kinase A

POLY I:C: polyinosinic: polycytidylic acid

M-CSF: macrophage colony-stimulating factor

SAL: SAL003

TGF: transforming growth factor

TNF: tumor necrosis factor

TUN: tunicamycin

VEGF: vascular endothelial growth factor

Supplemental Table 1

Donor#	Age	Gender	Death to Cultivation
1	56	M	6
2	57	M	7
3	61	F	6
4	65	F	5
5	50	M	6
6	40	F	5
7	54.8	M	5.8 Whole cornea used for figure 3
8	8.8	F	0.8

SUPPLEMENTAL Table 2

**Western blot antibodies for ISR pathway**				
	Primary Antibody	Conct.	Secondary Antibody	Conct.
**ATF4**	Recombinant Anti-ATF-4 antibody (ab184909, abcam, Boston, MA)	1:1000	Anti-rabbit IgG, HRP-linked antibody (#7074, Cell Signaling, Danvers, MA)	1:5000
**peIF2α**	Recombinant anti-EIF2S1 (phospho S51) antibody (ab 32157, abcam)	1:1000	Anti-rabbit IgG, HRP-linked antibody (#7074, Cell Signaling)	1:5000
**eIF2α**	eIF2α Antibody (#9722, Cell Signaling)	1:1000	Anti-rabbit IgG, HRP-linked antibody (#7074, Cell Signaling)	1:5000
**CHOP**	CHOP (L63F7) Mouse mAb (#2895, Cell Signaling)	1:1000	Anti-mouse IgG, HRP-linked antibody (#7076, Cell Signaling)	1:5000
**GAPDH**	Anti-GAPDH antibody, Mouse monoclonal (G8795, Sigma-Aldrich, St. Louis, MO)	1: 5000	Anti-mouse IgG, HRP-linked antibody (#7076, Cell Signaling)	1:5000

Supplemental Table 3

Miliplex Human Cytokine/Chemokine immunoassay (HCYTA-60K-PX48)	
sCD40L	IL-12 (p70)
EGF	IL-13
Eotaxin	IL-15
EGF-2	IL-17A
FLT-3L	IL-17E/IL-25
Fractalkine	IL-17F
G-CSF	IL-18
GM-CSF	IL-22
GROα	IL-27
IFNα2	IP-10
IFNγ	MCP-1
IL-1α	MCP-3
IL-1 β	M-CSF
IL-1RA	MDC
IL-2	MIG
IL-3	MIP-1α
IL-4	MIP-1 β
IL-5	PDGF-AA
IL-6	PDGF-AB/BB
IL-7	RANTES
IL-8	TGFα
IL-9	TNFα
IL-10	TNF β
IL-12(p40)	VEGF-A

Supplemental Figure 1 – Generation of CHOP-/- cell line.

Target Gene: DDIT3 : ENST00000552740 (primary) (-)

Target sequence: >12 dna:chromosome chromosome:GRCh38:12:57515988:57522337:-1

4801 CTTTACCTACAAAAACAGGCATCAGACCAGCTTGCCAACTTGTGGCATAGACTGTTTGCT 4860

4861 ACATGGAGCTTGTTCCAGCCACTCCCCATTATCCTGCAGATGTGCTTTTCCAGACTGATC 4920

4921 CAACTGCAGAGATGGCAGCTGAGTCATTGCCTTTCTCCTTCGGGACACTGTCCAGCTGGG 4980

4981 AGCTGGAAGCCTGGTATGAGGACCTGCAAGAGGTCCTGTCTTCAGATGAAAATGGGGGTA 5040

5041 CCTATGTTTCACCTCCTGGAAATGAAGAGGTAAGAATGTTAGCCCTAAAGCTAAAGGGGG 5100

5101 ATGTTACCTTTCCCTTCTCAACTAATATCTATGTTCCCTTTCCTCATTTCCTTGAAGGAA 5160

5161 GAATCAAAAATCTTCACCACTCTTGACCCTGCTTCTCTGGCTTGGCTGACTGAGGAGGAG 5220

5221 CCAGAACCAGCAGAGGTCACAAGCACCTCCCAGAGCCCTCACTCTCCAGATTCCAGTCAG 5280

5281 AGCTCCCTGGCTCAGGAGGAAGAGGAGGAAGACCAAGGGAGAACCAGGAAACGGAAACAG 5340

5341 AGTGGTCATTCCCCAGCCCGGGCTGGAAAGCAGCGCATGAAGGAGAAAGAACAGGAGAAT 5400

5401 GAAAGGAAAGTGGCACAGCTAGCTGAAGAGAATGAACGGCTCAAGCAGGAAATCGAGCGC 5460

5461 CTGACCAGGGAAGTAGAGGCGACTCGCCGAGCTCTGATTGACCGAATGGTGAATCTGCAC 5520

5521 CAAGCATGAACAATTGGGAGCATCAGTCCCCCACTTGGGCCACACTACCCACCTTTCCCA 5580

Green- Primer positions

Red -gRNA target

Yellow – SAC1 cleavage site

gRNA - AGATTCCAGTCAGAGCTCCC **TGG**

Agarose gel of CHOP -/- and WT clones to determine CRISPR targeting.

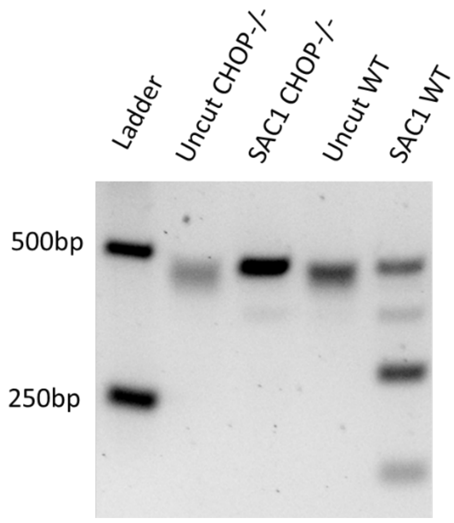

Supplemental Table 4, results from multiplex experiment

**Plate**	Plate 1	Plate 1	Plate 1	Plate 1	Plate 1	Plate 1	Plate 1	Plate 1	Plate 1	Plate 1
**Group**	Backgroun	Standard 1	Standard 2	Standard 3	Standard 4	Standard 5	Standard 6	Standard 7	Quality Co	Quality Control 2
**Well ID**	1	2	3	4	5	6	7	8	9	10
**Sample ID**	Backgroun	Standard1	Standard2	Standard3	Standard4	Standard5	Standard6	Standard7	Unknown3	Unknown40
**sCD40L**	-	17.06	68.14	305.75	1571.96	9617.84	46589.72	########	170.06	939.31
**EGF**	-	-	12.85	93.13	372.62	1969.32	6948.00	47739.50	65.28	615.80
**EOTAXIN**	-	2.45	15.40	92.84	397.09	1543.58	8731.95	47399.10	151.83	629.39
**FGF-2**	-	-	219.96	731.10	2537.73	10538.43	86962.49	########	652.63	3566.45
**FLT-3L**	-	0.55	5.53	38.66	233.26	1038.06	2465.63	13935.81	60.24	410.85
**FRACTALKI**	-	30.63	156.72	919.37	3663.57	13982.87	########	########	1283.61	4896.73
**G-CSF**	-	9.69	26.08	109.67	548.86	3924.05	18855.94	69755.55	175.53	1435.10
**GM-CSF**	-	-	18.43	99.92	293.89	1439.14	9181.99	38230.26	54.79	358.57
**GROa**	-	1.32	6.21	33.34	170.88	671.03	4004.72	18720.94	38.63	210.02
**IFNa2**	-	9.12	37.34	205.70	1039.53	4840.03	22040.69	########	282.15	1728.94
**IFNg**	-	-	6.98	33.50	148.78	877.60	4017.07	19549.98	90.35	483.85
**IL-1a**	-	4.49	25.54	139.67	545.57	2684.26	20046.00	75493.27	199.80	907.97
**IL-1b**	-	1.49	8.62	39.70	192.90	1161.08	4475.81	25493.04	55.99	337.29
**IL-1ra**	-	1.56	8.06	43.90	190.00	1075.24	4393.60	24023.18	66.32	410.74
**IL-2**	-	0.81	3.01	15.96	83.57	422.11	1681.01	9653.83	23.06	143.46
**IL-3**	-	-	10.46	44.91	149.89	455.09	3062.76	19447.41	15.13	82.01
**IL-4**	-	0.63	3.25	16.95	84.67	383.93	1659.28	9607.33	23.53	145.82
**IL-5**	-	0.62	3.07	17.76	79.09	439.00	1722.68	9788.85	29.09	163.74
**IL-6**	-	0.70	2.89	16.74	92.02	386.32	1654.92	9692.53	22.96	146.58
**IL-7**	-	-	7.58	23.56	81.69	366.04	1822.61	9728.66	24.20	137.73
**IL-8**	-	0.65	3.13	16.64	85.22	384.77	1719.65	9579.91	22.98	143.70
**IL-9**	-	0.61	4.17	15.72	77.19	506.47	1499.77	9281.66	23.00	168.16
**IL-10**	-	2.44	12.63	68.10	320.97	1752.93	6834.94	39104.34	120.35	802.23
**IL-12 (p40)**	-	6.87	31.82	164.36	791.13	4268.63	17743.97	########	242.39	1421.98
**IL-12 (p70)**	-	3.08	15.97	82.62	448.64	1742.65	8660.37	47795.13	130.57	752.58
**IL-13**	-	8.73	29.14	154.46	844.57	4315.89	15831.02	########	151.53	1093.42
**IL-15**	-	3.45	14.97	79.80	463.93	1871.99	7125.80	48286.01	79.50	574.26
**IL-17A**	-	1.58	7.08	30.39	151.25	931.41	4036.13	18997.93	50.74	348.45
**IL-17E/IL2!**	-	45.56	177.36	1170.27	4747.25	36816.91	68558.67	########	1739.61	8565.85
**IL-17F**	-	31.69	157.16	961.76	3538.54	15932.62	########	########	1454.73	5508.00
**IL-18**	-	0.67	3.13	16.05	86.60	386.99	1896.27	9502.70	25.59	140.72
**IL-22**	-	20.47	56.60	351.05	1769.79	8978.27	40088.95	########	442.89	2137.23
**IL-27**	-	26.77	77.29	408.68	2014.96	7418.65	########	########	562.42	2958.77
**IP-10**	-	0.81	13.61	186.24	997.17	2613.38	4988.77	37733.90	438.08	1408.84
**MCP-1**	-	3.52	14.33	91.35	403.68	1740.75	33951.42	45144.02	243.21	916.87
**MCP-3**	-	2.50	16.65	89.24	337.25	1618.96	20678.53	47256.64	154.92	528.72
**M-CSF**	-	39.75	204.40	1047.30	4970.67	24021.92	########	########	1555.42	9106.83
**MDC**	-	0.71	3.12	16.48	83.26	426.90	1509.70	9495.13	33.49	190.25
**MIG**	-	6.35	30.95	164.67	824.98	3856.04	20891.65	95432.53	318.72	2139.45
**MIP-1a**	-	0.42	1.92	9.70	47.88	279.13	784.14	5736.11	20.45	85.59
**MIP-1b**	-	-	2.24	9.24	51.22	207.50	628.22	5686.14	15.94	90.43
**PDGF-AA**	-	10.59	43.12	287.72	1111.23	6673.89	19233.22	########	533.67	2703.60
**PDGF-AB/I**	-	-	66.82	303.52	1866.73	16121.47	########	########	421.43	3967.62
**RANTES**	-	1.39	6.27	31.64	164.24	823.51	4181.12	18441.44	49.77	300.58
**TGFa**	-	1.21	6.38	35.01	158.64	730.50	1459.85	19859.26	51.00	281.41
**TNFa**	-	6.89	30.92	159.36	813.69	4977.05	18526.82	91357.61	241.05	1385.13
**TNFb**	-	1.59	8.34	41.14	183.75	1147.50	4911.13	24176.58	58.81	316.41
**VEGF-A**	-	1.98	12.55	70.81	320.31	1617.45	7480.18	39079.58	123.01	787.95

	Ctrl 1	Ctrl 2	Ctrl 3	Ctrl 4	Tun 1	Tun 2	Tun 3	Tun 4	Sal 1	Sal 2	Sal 3	Sal 4
**sCD40L**	7.98	5.55	14.15	-	11.49	-	7.51	-	11.79	7.74	-	-
**EGF**	-	-	-	-	-	-	-	-	-	-	-	-
**EOTAXIN**	-	-	-	-	-	-	-	-	-	-	-	-
**FGF-2**	-	-	-	-	-	-	-	-	-	-	-	-
**FLT-3L**	0.37	0.44	0.59	0.40	0.29	0.22	0.34	0.29	0.22	0.45	0.42	0.17
**FRACTALKI**	11.95	-	15.59	-	15.48	-	8.02	-	10.44	10.32	-	-
**G-CSF**	2824.17	3027.86	9991.53	2042.86	1977.14	1558.54	2751.11	853.38	3974.58	2547.70	3920.03	1472.67
**GM-CSF**	-	-	-	-	-	-	-	-	-	0.24	-	-
**GROa**	86.28	65.01	207.55	34.62	64.50	24.35	28.50	13.27	63.05	33.26	39.47	15.82
**IFNa2**	-	-	-	-	-	-	-	-	-	-	-	-
**IFNg**	-	-	-	-	-	-	-	-	-	-	-	-
**IL-1a**	212.49	222.03	437.07	178.29	357.70	169.41	194.10	86.70	271.66	174.68	173.46	87.95
**IL-1b**	1.04	0.57	0.57	-	1.14	-	0.36	-	0.93	0.83	0.57	-
**IL-1ra**	8.26	7.50	6.89	6.26	10.05	6.95	2.34	4.51	7.25	6.82	2.67	4.20
**IL-2**	-	-	-	0.12	0.15	-	-	-	-	-	-	-
**IL-3**	-	-	-	-	-	-	-	0.84	1.21	0.38	2.45	-
**IL-4**	0.14	0.03	0.25	0.15	1.66	1.36	1.54	1.02	0.86	1.07	1.30	0.37
**IL-5**	-	-	0.07	-	-	-	-	-	-	-	-	-
**IL-6**	8.43	2.98	23.10	0.93	42.91	8.83	16.17	2.35	17.45	5.05	9.94	1.11
**IL-7**	-	-	-	-	-	-	-	-	-	-	-	-
**IL-8**	44.99	30.87	117.69	19.43	106.46	27.29	30.03	15.32	86.12	30.56	31.18	13.66
**IL-9**	1.29	0.44	2.23	1.00	1.63	0.44	1.12	0.60	1.39	1.63	1.48	-
**IL-10**	1.00	0.72	1.40	0.56	1.00	0.35	0.89	0.45	0.89	0.97	1.00	-
**IL-12 (p40)**	-	-	2.10	-	2.41	-	-	-	-	-	-	-
**IL-12 (p70)**	-	-	-	0.11	0.29	-	-	-	-	-	-	-
**IL-13**	-	-	-	-	1.92	0.87	1.27	1.03	-	0.62	0.87	-
**IL-15**	0.62	0.41	0.80	0.45	0.57	0.32	0.50	0.32	0.32	0.49	0.35	-
**IL-17A**	-	-	-	-	-	-	-	-	-	-	-	-
**IL-17E/IL2!**	3.74	2.56	6.23	3.85	6.22	1.15	3.43	3.24	3.24	5.23	5.41	-
**IL-17F**	0.27	-	0.52	-	0.43	-	-	-	0.32	-	-	-
**IL-18**	2.69	1.34	1.74	0.83	2.05	1.21	0.74	0.90	1.60	0.75	0.83	1.80
**IL-22**	21.14	14.28	16.94	17.52	26.11	17.74	18.82	22.02	16.17	23.64	25.13	8.38
**IL-27**	35.38	-	36.59	31.09	45.85	-	-	30.06	19.56	33.30	31.04	-
**IP-10**	486.36	426.05	1195.92	452.80	452.99	99.69	58.63	132.85	303.03	119.61	178.65	124.17
**MCP-1**	-	-	-	-	-	-	-	-	-	-	-	-
**MCP-3**	0.82	0.18	0.72	0.84	0.92	0.35	0.80	0.69	0.38	1.13	1.43	-

	Average Ct	Average Tu	Average Sal
**sCD40L**	9.23	9.50	9.77
**EGF**	#DIV/0!	#DIV/0!	#DIV/0!
**EOTAXIN**	#DIV/0!	#DIV/0!	#DIV/0!
**FGF-2**	#DIV/0!	#DIV/0!	#DIV/0!
**FLT-3L**	0.45	0.29	0.32
**FRACTALKI**	13.77	11.75	10.38
**G-CSF**	4471.61	1785.04	2978.74
**GM-CSF**	#DIV/0!	#DIV/0!	0.24
**GROa**	98.37	32.66	37.90
**IFNa2**	#DIV/0!	#DIV/0!	#DIV/0!
**IFNg**	#DIV/0!	#DIV/0!	#DIV/0!
**IL-1a**	262.47	201.98	176.94
**IL-1b**	0.73	0.75	0.78
**IL-1ra**	7.23	5.96	5.23
**IL-2**	0.12	0.15	#DIV/0!
**IL-3**	#DIV/0!	0.84	1.35
**IL-4**	0.14	1.40	0.90
**IL-5**	0.07	#DIV/0!	#DIV/0!
**IL-6**	8.86	17.56	8.39
**IL-7**	#DIV/0!	#DIV/0!	#DIV/0!
**IL-8**	53.25	44.78	40.38
**IL-9**	1.24	0.95	1.50
**IL-10**	0.92	0.67	0.96
**IL-12 (p40)**	2.10	2.41	#DIV/0!
**IL-12 (p70)**	0.11	0.29	#DIV/0!
**IL-13**	#DIV/0!	1.27	0.74
**IL-15**	0.57	0.43	0.39
**IL-17A**	#DIV/0!	#DIV/0!	#DIV/0!
**IL-17E/IL2!**	4.09	3.51	4.63
**IL-17F**	0.39	0.43	0.32
**IL-18**	1.65	1.23	1.24
**IL-22**	17.47	21.17	18.33
**IL-27**	34.35	37.96	27.96
**IP-10**	640.28	186.04	181.36
**MCP-1**	#DIV/0!	#DIV/0!	#DIV/0!
**MCP-3**	0.64	0.69	0.98

**M-CSF**	46.76	48.06	75.50	39.49	105.63	91.01	121.84	76.02	30.66	45.85	48.06	17.44
**MDC**	0.12	0.03	-	0.02	0.11	0.05	0.14	0.05	0.09	0.27	0.99	-
**MIG**	4.37	2.62	7.70	2.88	5.24	1.81	3.06	1.63	3.76	3.23	4.71	-
**MIP-1a**	0.09	0.63	-	-	-	-	0.79	-	-	-	1.42	-
**MIP-1b**	0.61	-	0.74	-	0.82	-	-	-	0.40	-	-	-
**PDGF-AA**	367.02	380.09	439.76	461.34	271.27	332.10	379.49	351.99	192.56	408.01	420.24	288.05
**PDGF-AB/I**	-	-	13.26	-	36.33	-	-	-	-	-	-	-
**RANTES**	2.16	2.26	4.71	1.87	3.09	2.81	5.09	1.72	4.10	6.29	20.66	2.15
**TGFa**	0.83	0.35	0.79	0.54	1.61	0.76	0.76	0.81	0.61	0.79	0.83	-
**TNFa**	3.92	3.75	8.72	2.91	5.03	2.71	3.62	1.99	5.49	4.86	6.55	2.21
**TNFb**	0.83	0.13	1.15	0.42	4.78	3.70	4.33	2.75	2.63	2.98	3.50	0.79
**VEGF-A**	38.89	27.96	71.79	17.60	442.03	423.72	525.91	332.92	299.85	415.87	573.78	194.83

**M-CSF**	52.45	98.63	35.50
**MDC**	0.06	0.09	0.45
**MIG**	4.39	2.93	3.90
**MIP-1a**	0.36	0.79	1.42
**MIP-1b**	0.68	0.82	0.40
**PDGF-AA**	412.05	333.71	327.22
**PDGF-AB/I**	13.26	36.33	#DIV/0!
**RANTES**	2.75	3.18	8.30
**TGFa**	0.63	0.99	0.74
**TNFa**	4.82	3.34	4.78
**TNFb**	0.63	3.89	2.47
**VEGF-A**	39.06	431.14	371.08

Supplemental Figure 2.
ISR induction in resposne to SAL003 overnight challenge from Figure 1, scale bar 50μm.

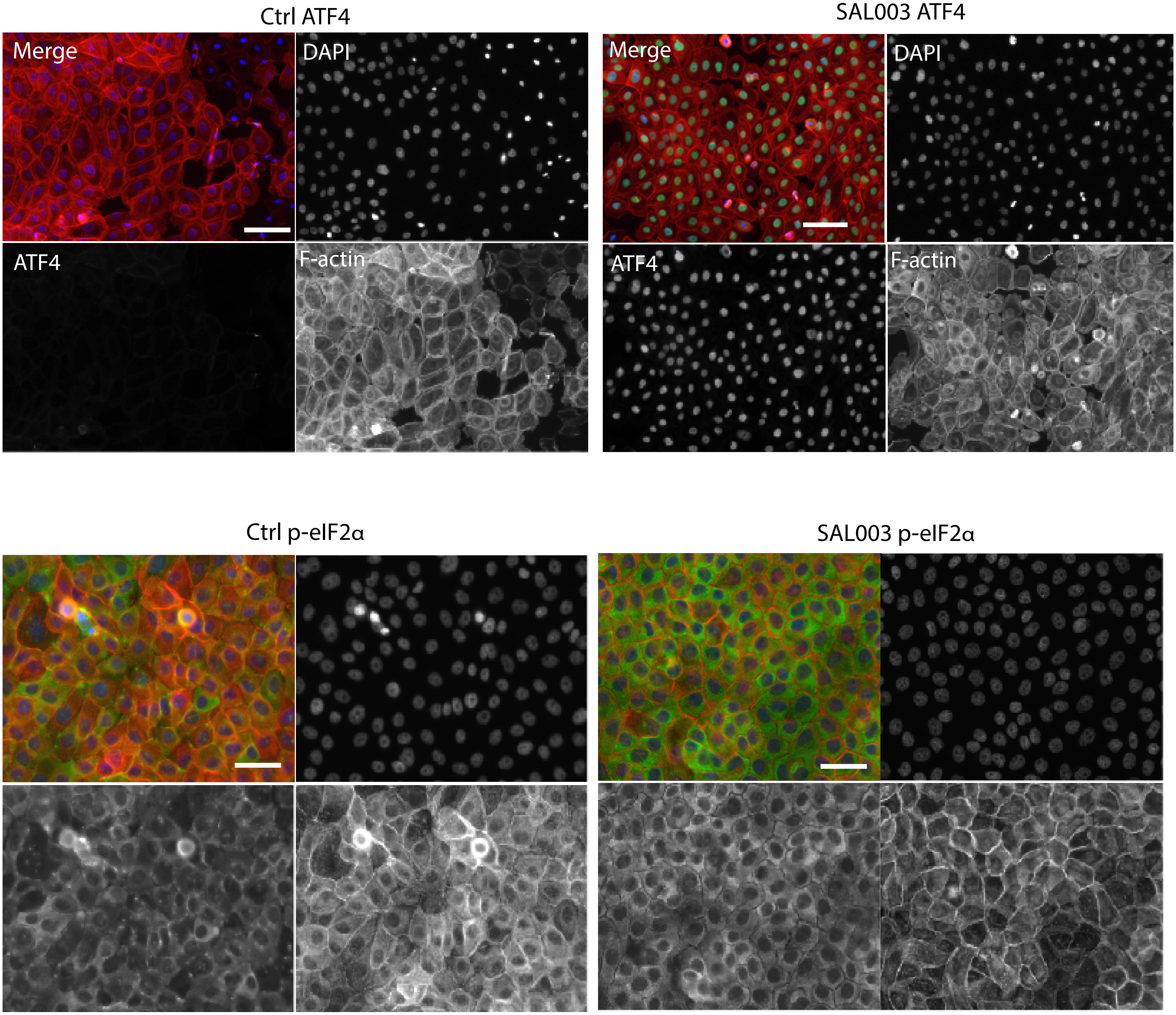

Supplemental Figure 3. Activation of the CHOP after 16 hours incubation with ISR aonists

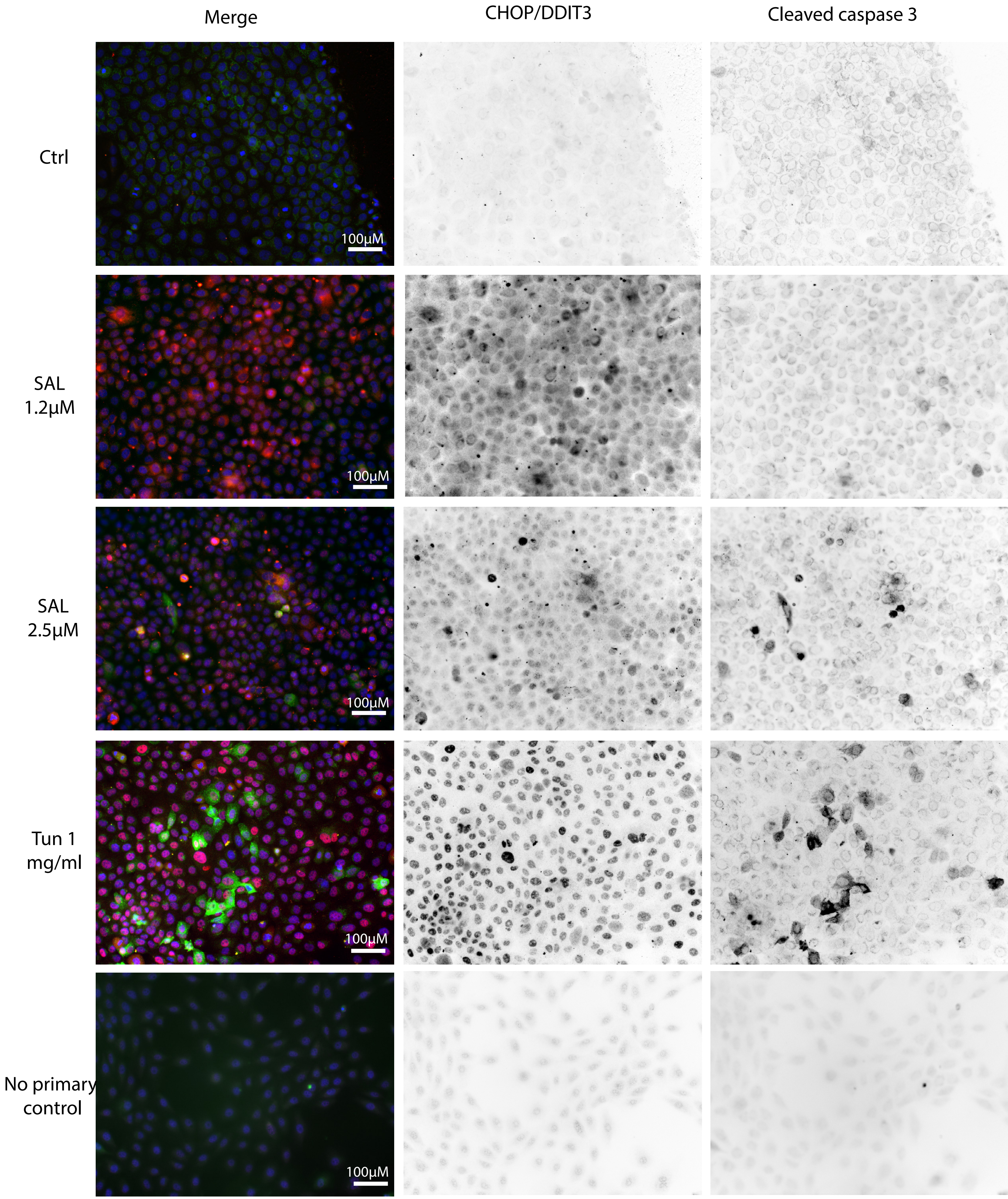

Supplemental figure 4. XBP1-sp reporter line in response to scratch assay.

Stitched images of XBP1-1 (green), DAPI (Blue), after scratch assay at 0-6 hours.
Graph shows relative (to time 0) green fluorescence. Tunicamycin as positive control.

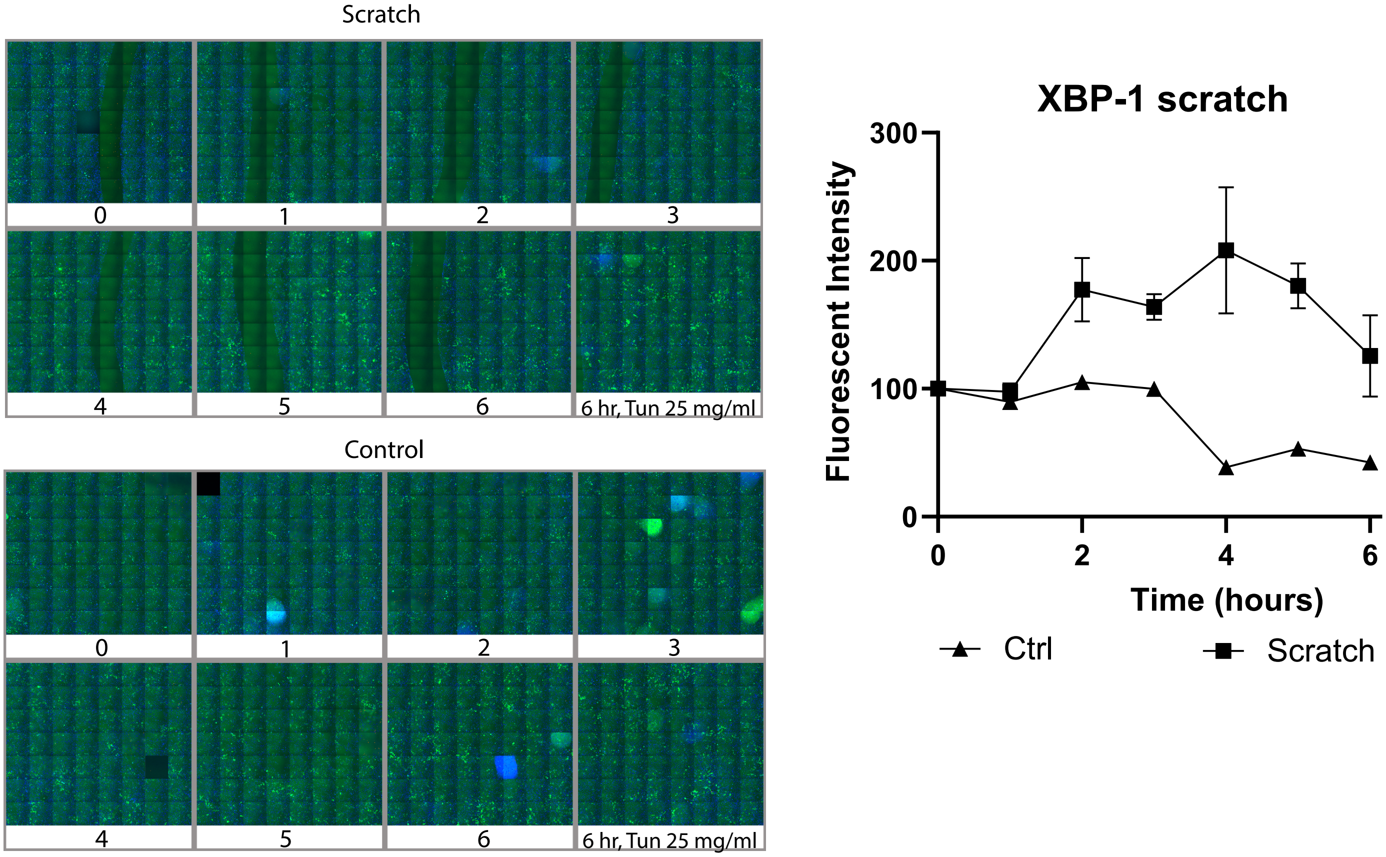

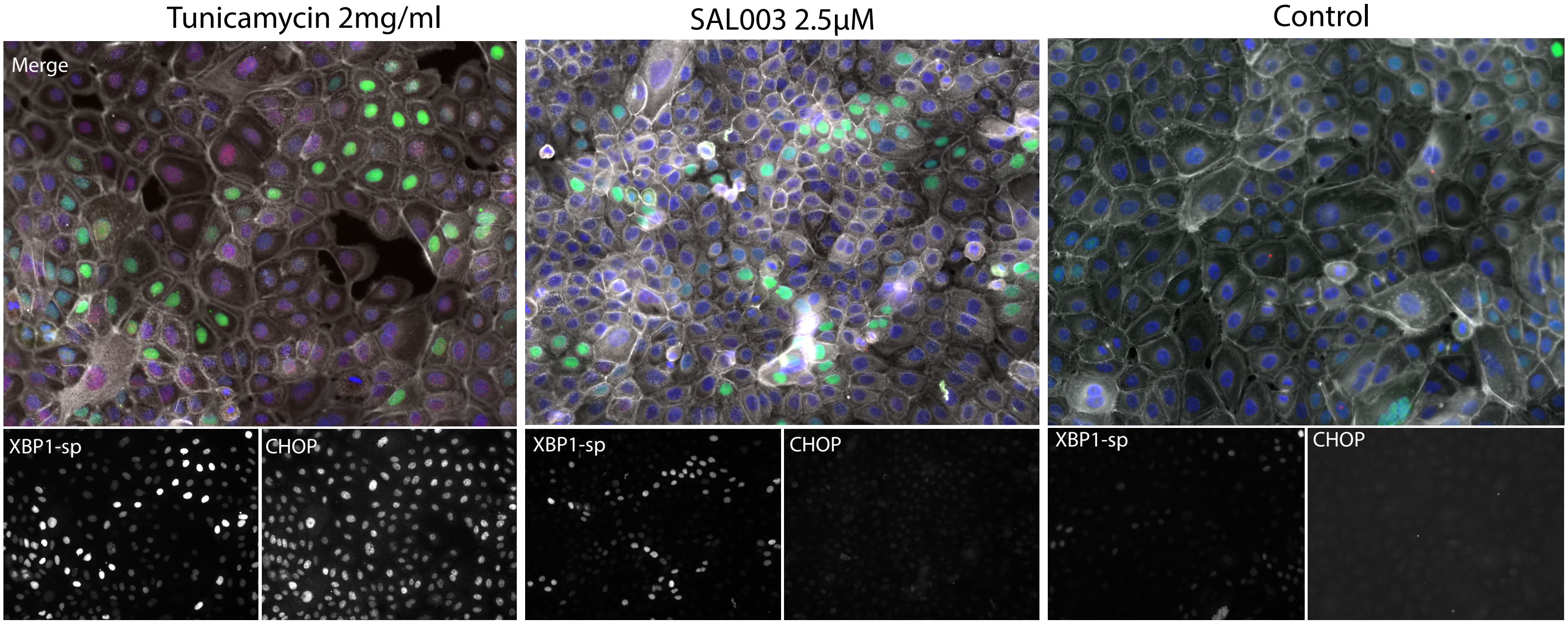

Induction of XBP-1 (green) and CHOP (red) in response to ISR challenge over 16 hours, DAPI-Blue.
